# Comparative Analysis of Serum BAFF and IL-17 Levels Pre- and Post-Antipsychotic Treatment for Acute Schizophrenia

**DOI:** 10.3390/ijms26010385

**Published:** 2025-01-04

**Authors:** Samar Samoud, Ahlem Mtiraoui, Imen Zamali, Yousr Galai, Naila Hannachi, Wiem Manoubi, Jaafar Nakhli, Hechmi Louzir, Yousri El Kissi

**Affiliations:** 1 Department of Clinical Immunology, Institut Pasteur de Tunis, Tunis 1002, Tunisia; imen.zamali@fmt.utm.tn (I.Z.); yousr.galai@pasteur.tn (Y.G.); 2 Faculty of Medicine of Sousse, University of Sousse, Sousse 4002, Tunisia; ahlamtiraoui@yahoo.fr (A.M.); naila.hannachi@rns.tn (N.H.); nakhlijaaf@yahoo.fr (J.N.); 3 Laboratory of Transmission, Control and Immunobiology of Infections (LR16IPT02), Institut Pasteur de Tunis, Tunis 1002, Tunisia; hechmi.louzir@pasteur.tn; 4 Department of Psychiatry, Farhat Hached University Hospital, Sousse 4000, Tunisia; 5 Research Laboratory LR12ES04, Faculty of Medicine of Sousse, University of Sousse, Sousse 4002, Tunisia; yousri.elkissi@yahoo.com; 6 Faculty of Medicine of Tunis, University of Tunis El Manar, Tunis 1068, Tunisia; 7 Faculty of Pharmacy, University of Monastir, Monastir 5000, Tunisia; 8 Department of Neuroscience, Erasmus University Medical Centre, 3000 GD Rotterdam, The Netherlands; wiem.manoubi@yahoo.fr; 9 Research Laboratory of Human Cytogenetics, Molecular Genetics and Reproductive Biology LR03SP02, Farhat Hached University Hospital, Sousse 4000, Tunisia

**Keywords:** schizophrenia, cytokines, interleukin-17, B cell-activating factor, antipsychotics

## Abstract

The interplay between the cytokine network and antipsychotic treatment in schizophrenia remains poorly understood. This study aimed to investigate the impact of psychotropic medications on serum levels of IFN-γ, IL-4, TGF-β1, IL-17, and BAFF, and to explore their relationship with psychopathological features. We recruited 63 patients diagnosed with schizophrenia in the acute phase, all of whom were either drug-naïve or had been drug-free for at least three months. Serum levels of IL-4, IFN-γ, TGF-β1, IL-17, and BAFF were measured at baseline and after six months of antipsychotic treatment. The severity of symptoms was assessed using the Brief Psychiatric Rating Scale (BPRS), the Scale for the Assessment of Positive Symptoms (SAPS), and the Scale for the Assessment of Negative Symptoms (SANS). Fifty-two patients completed the six-month follow-up for immunoassay analysis. Antipsychotic treatment led to a significant decrease in serum levels of IFN-γ, TGF-β1, and IL-17, alongside a significant increase in BAFF levels. Changes in IFN-γ were positively correlated with SANS scores and negatively correlated with Global Assessment of Functioning (GAF) scores. Changes in TGF-β1 were negatively correlated with GAF scores. Changes in BAFF were negatively correlated with SAPS scores. Multivariable regression models were used to explore the association between cytokine level changes (IL-17, BAFF, IFN-γ, and TGF-β1) and independent variables, including demographic (gender, age), behavioral (tobacco use), clinical (schizophrenia type, disease course, date of onset, prior treatment), and biological (C-reactive protein (CRP), erythrocyte sedimentation rate (ESR)) factors, as well as standardized assessment scores. No significant associations were found, except for a significant negative correlation between TGF-β1 changes and GAF scores, as well as a positive correlation with age. Interestingly, advanced statistical analyses revealed that only changes in IL-17 and BAFF levels were significantly associated with antipsychotic treatment. Our findings suggest that antipsychotic drugs exert both pro- and anti-inflammatory effects on the cytokine network. The observed modulation of IL-17 and BAFF highlights their potential as future therapeutic targets in schizophrenia.

## 1. Introduction

Although the pathological mechanisms of schizophrenia still need to be elucidated, several lines of evidence over the last decades have implicated the immune system and the inflammatory mediators in the etiopathogenesis of the disease [1,2,3,4]. Within this context, cytokine alterations have been extensively investigated [5,6,7,8,9,10,11,12]. It has been suggested that pro-inflammatory cytokines’ augmentation in the central nervous system can lead to impaired brain function [13]. Increased levels of TNF-α, IL-1, IL-2, IL-6, and IFN-γ have been repeatedly observed [11,14,15,16]. IL-17, a leading pro-inflammatory cytokine, has been poorly investigated in schizophrenia patients and studies provided conflicting results [17,18,19,20,21,22]. The role of some anti-inflammatory cytokines as immunoregulatory molecules, such as IL-4 and TGF-β1, has also been examined [22,23,24,25].

There is an emerging literature reporting an interaction between cytokine network and antipsychotic treatment for acute and chronic schizophrenia patients [4,26,27,28,29,30,31,32,33].

Some authors proposed that the clinical efficacy of antipsychotics might be related to their immunosuppressive effects and their capacity to normalize pro-inflammatory immune changes [28,31,34]. Although not all studies yielded the same conclusions, it has been suggested that alterations in the immune system might be more pronounced in treatment-resistant schizophrenia patients [27,35,36]. Further data attributed the efficacy of Clozapine and Olanzapine to their pro-inflammatory effects [37,38]. The interpretation of the association remains, nonetheless, speculative. The paucity of data and the wide discrepancies of clinical contexts and study variables may account for the divergent results.

Yet, there is a lack of literature regarding the effects of antipsychotics on a large set of cytokines. Data related to IL-17 are scarce and inconsistent [18,19,21,22,37,39,40]. Alterations in this pathway may play a role in the development of schizophrenia symptoms [19,22]. In fact, the IL-17 receptor is expressed in many neural tissues and both neural damaging and beneficial effects of the cytokine have been described [41,42]. Their mouse model showed that increased, infection-induced levels of peripheral IL-17A in the mother caused irregularities in the neuron layers of the brain cortex in offspring, and these were associated with deficits in social interaction, behavior, and communication [41]. Meanwhile, the offspring of mice who lacked an essential regulator of the IL-17 pathway did not show these behavioral deficits [41]. In addition, Vergaelen et al. showed an increased Th17 percentage in adults with psychotic symptoms as compared to non-psychotic adults, and the Th17 percentage was related to the presence of positive psychotic symptoms [43]. Regarding the circulating levels of IL-17, opposite results have been published [21,42,44]. Only a few studies focused on antipsychotic drugs’ effects on IL-17, with inconsistent results.

B cell-activating factor (BAFF), belonging to the TNF family, has been implicated in many autoimmune and inflammatory diseases with neurological symptoms [45,46,47,48]. An association between IL-17 and BAFF serum levels has already been stated. BAFF may increase IL-6 receptor expression on CD4+T cells, enhance IL6-IL-6R/p-STAT3 signaling pathway, and promote dendritic cell maturation and function [49]. IL-6/IL-6R signal pathway is important for Th17 cell differentiation and would play a crucial role in the expansion of Th17 cells [50,51]. To clarify the effects of antipsychotics on BAFF levels would be of great interest. However, to the best of our knowledge, no previous study focused on antipsychotic drugs’ effects on BAFF serum levels.

Altogether, these considerations enhance the importance of examining prospectively the effects of antipsychotics on the cytokine network of patients suffering from schizophrenia.

The aim of our study was (1) to assess prospectively serum levels of IFN-γ, IL-4, and IL-17 representative of type-1, type-2, and type-17 cytokines and the regulatory cytokines TGF-β1 and BAFF in drug-free schizophrenia patients in the acute phase of the disease and after antipsychotic treatment and (2) to determine correlations between cytokine levels changes and clinical and psychopathological features.

We tested the following hypotheses: (1) antipsychotic treatments influence serum levels of IFN-γ, IL-4, IL-17, TGF-β1, and BAFF in schizophrenia patients; (2) modulating effect of antipsychotics would be either anti-inflammatory or pro-inflammatory; and (3) changes in cytokine serum levels are correlated to psychopathological features.

## 2. Results

### 2.1. Demographic and Clinical Characteristics

The patients’ demographic and clinical characteristics are summarized in Table 1.

Data from standardized assessments are presented in Table 2.

### 2.2. Circulating Cytokine Serum Levels

Six months after appropriate antipsychotic therapy, patients exhibited significantly decreased serum levels of IFN-γ, TGF-β1, and IL-17 compared to baseline (*p* < 10^−3^). BAFF levels were significantly increased (*p* = −0.017). Levels of IL-4 were too low for detection in the acute phase of the disease as well as after antipsychotic treatment. Results for assays of cytokines are summarized in Table 3. 

The data did not follow a normal distribution, as confirmed by the Z de Kolmogorov–Smirnov non-parametric test. Serum cytokine levels were analyzed using the Wilcoxon non-parametric test, while the Z-test was employed to evaluate cytokine markers associated with the drug’s effects. The results revealed a significant association between IL-17 and BAFF levels and the treatment (Table 4).

### 2.3. Correlations Between Changes in Cytokine Serum Levels and Clinical Features

There were no statistically significant correlations between changes in cytokine levels and any clinical variable, including patient’s present age, age of illness onset, duration of the illness and duration of untreated psychosis.

As represented in Table 5, changes in IFN-γ serum levels were correlated positively with the SANS score (r = 0.39; *p* = 0.003) and negatively with the GAF score (r = −0.45; *p* < 10^−3^). Changes in TGF-β1 serum levels were correlated negatively with the GAF score (r = −0.27; *p* = 0.04). BAFF changes were negatively correlated with changes in the SAPS score (r = −0.34; *p* = 0.01).

Using, multivariable regression models to establish the relationship between the major dependent variables (variation in IL17, BAFF, INF-γ, and TGF-β1) and the score variation, only correlation between the level of INF difference and the score variation in GAF and between the level of TGF-β1 difference and the score variation in GAF were confirmed (Table 6).

### 2.4. Correlations Between Changes in Cytokine Serum Levels and Demographic, Behavior, Clinical, and Biological Independent Variables

Multivariable regression models are used to establish the relationship between the major dependent variables (variation in IL-17, BAFF, INF-γ, and TGF-β1) and demographic (gender and age), behavior (tobacco use, BMI has not been calculated), clinical (schizophrenia type and evolution, date of onset of the disease, previous treatment), and biological (ESR and CRP) independent variables.

The variation was not significantly associated with all variables included in the models, with the exception of the significant association found between Δ TGF-β1 and age (Table 7).

## 3. Discussion

In this study, IL-4 levels in schizophrenia patients were too low for detection at baseline and remained unchanged after six months of antipsychotic treatment. This result is unlikely to be attributed to inappropriate serum storage conditions or issues with the cytokine assay, as the calibration curve was valid and other cytokines were successfully detected. Previous research has suggested that antipsychotic medications can suppress certain type-2 cytokines, including IL-4 [24,39] and IL-6 [52]. While some studies have reported a significant increase in IL-4 serum levels following treatment [34,53,54,55], others have observed unchanged levels after antipsychotic treatment, as supported by the meta-analysis of Tourjman et al. [38]. Conversely, Rapaport and Miller reported a significant decrease in IL-4 levels following treatment of acute psychosis in first-episode psychosis patients and those with chronic schizophrenia [56]. Our findings align with the meta-analysis of Tourjman et al., which strengthens the evidence that IL-4 serum levels remain stable after antipsychotic treatment. Our findings are also consistent with those of Kim et al., who detected IL-4 in only 20.5% of patients, despite their larger sample size (88 patients) and higher assay efficiency. The absence of IL-4 detection in our study, as well as in that of Kim et al., may be explained by the selection of patients in the acute phase of the disease who were not receiving treatment. However, the same authors reported a significant increase in IL-4 levels after patients started neuroleptic treatment [57], which is not our case. Further studies would be desirable to provide more answers.

Our study also revealed a significant decrease in serum levels of IFN-γ, TGF-β1, and IL-17 following six months of antipsychotic treatment. The impact of antipsychotic drugs on IFN-γ levels remains controversial. Some authors reported no changes during treatment [41], while others, including our study, have found a reduction in IFN-γ levels following antipsychotic treatment [34,58]. According to the meta-analysis by Rapaport and Miller, chronically ill patients exhibit significantly lower IFN-γ levels compared to healthy controls, with no significant changes observed after antipsychotic treatment [56]. Our findings support the hypothesis that antipsychotic drugs may reduce IFN-γ levels, although further studies are needed to clarify the mechanisms underlying this effect.

Regarding TGF-β1, previous research suggests that antipsychotics do not significantly affect its serum levels [38]. Our study observed a reduction in TGF-β1 levels after antipsychotic treatment, but the existing literature remains inconclusive. More studies with larger sample sizes are required to determine the consistency of this finding. One of the most important findings of our study was the significant decrease in IL-17 serum levels after antipsychotic treatment. IL-17 has been poorly studied in schizophrenia, and the available data on the effects of antipsychotic drugs on IL-17 levels are limited and contradictory. Himmerich et al. demonstrated an increase in IL-17 serum levels in healthy female subjects under the influence of antipsychotic drugs, which they attributed to a potential stimulation of type-17 immune responses [59]. However, these results were obtained using an in vitro stimulation model, which may not fully reflect in vivo conditions. Our study provides strong in vivo evidence of a decrease in IL-17 serum levels following treatment, supporting the idea that IL-17 may act as a state marker of acute schizophrenia, which normalizes with antipsychotic treatment. This hypothesis is consistent with Dimitrov et al., who found reduced IL-17 levels in patients with chronic paranoid schizophrenia under treatment [60]. Our previous work also reported higher IL-17 levels in drug-free schizophrenia patients compared to healthy controls, suggesting that elevated IL-17 may be an intrinsic feature of the disease itself [21].

Another key finding of our study was the significant increase in BAFF serum levels six months after antipsychotic treatment. To the best of our knowledge, this is the first study to specifically investigate the impact of antipsychotic drugs on BAFF levels in schizophrenia patients. Our findings are consistent with those of Ermakov et al., who observed elevated BAFF levels in schizophrenia patients undergoing treatment, but their study compared BAFF levels with healthy controls rather than assessing within-patient changes before and after treatment [61]. Engh et al. also compared treated schizophrenia patients with healthy controls but found no significant differences in BAFF levels between the two groups [62].

Interestingly, in our previous research, we identified a negative correlation between BAFF and IL-17 levels in antipsychotic-free patients [21], suggesting a potential coordinated regulation of these molecules. This observation is supported by previous studies highlighting a similar negative correlation between BAFF and IL-17, potentially mediated by Act1 adaptor protein, a key transcriptional regulator, in autoimmune diseases such as systemic lupus erythematosus and primary Sjögren’s syndrome [63,64]. The modulation of BAFF observed in this study, together with the decrease in IL-17, suggests that antipsychotic drugs may exert both pro- and anti-inflammatory effects on the cytokine network.

The correlation analysis in our study showed that changes in IFN-γ, TGF-β1, IL-17, and BAFF were associated with clinical improvement, but further statistical refinement revealed that only IL-17 and BAFF showed significant associations. This observation reinforces the hypothesis that IL-17 and BAFF play crucial roles in the psychopathological changes observed in schizophrenia. While the literature on cytokine changes in relation to symptom improvement is sparse, our findings are consistent with the results of De Witte et al., who found that only IL-10 levels decreased with symptom improvement following antipsychotic treatment [65,66,67,68,69]. Given the scarcity of evidence on these correlations and the heterogeneity of the findings, it is difficult to draw firm conclusions regarding the role of cytokines as direct mediators of treatment response. Nevertheless, the involvement of cytokines in the pathogenesis of schizophrenia is strongly supported, particularly through their role in inflammatory processes and the potential contribution of neuroinflammation to disease progression.

Our findings have important clinical implications. They suggest that antipsychotic drugs exert a modulatory effect on cytokines, which may be anti-inflammatory or pro-inflammatory depending on the specific cytokine target. For instance, while IL-17 levels decreased, BAFF levels increased, highlighting the complex and dynamic nature of cytokine regulation in response to treatment. This complexity calls into question the previously proposed potential of anti-inflammatory drugs as adjunctive therapies in schizophrenia [70]. Our study also highlights the potential of IL-17 and BAFF as future therapeutic targets for schizophrenia. Their modulation could serve as biomarkers of disease state or treatment response, as well as potential targets for novel therapeutic strategies aimed at improving patient outcomes.

Furthermore, we investigate the relationship between cytokine level changes (IL-17, BAFF, IFN-γ, and TGF-β1) and independent variables, including demographic (gender, age), behavioral (tobacco use), clinical (schizophrenia type, disease course, disease onset, prior treatment), and biological (CRP, ESR) factors, as well as standardized assessment scores. No significant associations were observed, except for a negative correlation between changes in TGF-β1 levels and GAF scores, and a positive correlation with age.

The major strengths of this study lie in its prospective design and the use of stringent inclusion criteria to minimize confounding factors. The comparison of cytokine levels within the same group of patients before and after treatment is a notable advantage, as it reduces inter-individual variability and provides a robust assessment of treatment effects. Furthermore, the use of standardized multiplex immunoassay analyses on antipsychotic-free patients followed prospectively enhances the validity of the findings.

However, some limitations should be acknowledged. First, the sample size of schizophrenia patients was relatively small, which may have limited the statistical power to detect subtle changes in cytokine levels. Additionally, substantial variability in serum interleukin levels was observed, suggesting heterogeneity in the cytokine response among patients. This variability could be related to differences in patient-specific characteristics, such as genetic background or environmental exposures. Future studies should aim to include larger cohorts and stratify patients based on relevant clinical and biological factors.

## 4. Materials and Methods

### 4.1. Subjects

Sixty-three patients with schizophrenia (DSM criteria) [71] were recruited shortly after being admitted to closed wards of the Department of Psychiatry, Farhat Hached Hospital of Sousse, Tunisia. At the time of inclusion, all of them were in an acute phase of the illness according to the Brief Psychiatric Rating Scale scores (BPRS ≥ 40). They were either drug-naïve or had been off medication for at least three months.

Patients with a history of autoimmune diseases, current infections, allergies, severe cognitive impairments, or other mental disorders significantly impairing communication were excluded. Initially, patients were assessed during the acute phase, and blood samples were collected for immunological analysis. Six months after starting treatment, all enrolled patients were contacted for follow-up. However, only 52 were eligible for the prospective immunoassay, as 13 participants were lost to follow-up or had not adhered to treatment.

All participants provided informed consent after a detailed explanation of the procedure. The study adhered to the principles of the Declaration of Helsinki and was approved by the Institutional Ethical Committee of the Faculty of Medicine “Ibn El-Jazzar” in Sousse, Tunisia (CEFMS 273/2024).

### 4.2. Assessment

A trained clinical psychiatrist assessed all participants. The diagnosis of schizophrenia was established based on the DSM criteria. Demographic data, medical history, and co-morbid disorders were collected. Symptoms severity and socio-occupational functioning were assessed using the Brief Psychiatric Rating Scale (BPRS) [72], the Positive and Negative Syndrome Scale (PANSS) [73], the Scale for the Assessment of Negative Symptoms (SANS) [60], the Scale for the Assessment of Positive Symptoms (SAPS) [74], and the Global Assessment of Functioning (GAF) [75].

### 4.3. Blood Sampling and Immunological Measures

For each participant, a blood sample was collected and forwarded to the Immunology Department of the hospital for immunological assay. Ten milliliters of fasting blood were withdrawn with an anticoagulant-free vacuum tube and centrifuged at 3800 rpm for 10 min. Sera were kept stored at −80 °C until thawed for assay.

The cytokine assays for IL-17, IFN-γ, TGF-β1, IL-4 and BAFF were performed using DuoSet ELISA Development System (R&D Systems, Minneapolis, MN, USA). IL-4 ELISA was established with a detection range from 3.0 to 1000 pg/mL using recommended buffers, diluents, and substrates. The coefficients of intra- and inter-assay variation were, respectively, 5.6% and 7.08%. IFN-γ ELISA was established with the detection range from 2 to 500 pg/mL in the same manner. The coefficients of intra- and inter-assay variation were, respectively, 6.7% and 9.2%. TGF-β1ELISA was established with a detection range of 50–2500 pg/mL and an assay was performed on the 12 times diluted activated plasma samples. Plasma samples were activated with an acidic solution since TGF-β1 is mainly secreted in latent forms [76]. The coefficients of intra- and inter-assay variation were 5.3% and 9.8%. The concentrations of the samples in each plate were calculated according to each standard curve and the dilution factor.

### 4.4. Statistical Analysis

Data were processed using SPSS version 17.0 software. A large variability of serum cytokine levels within the normal human population is already assumed. The application of Kolmogorov–Smirnov tests indicated that none of the measured cytokine values were normally distributed (*p* < 0.05). A non-parametric test (Wilcoxon test) has been performed to compare cytokines serum levels before and after applied therapy.

Spearman’s correlation evaluated the possible relationship between the cytokine changes and clinical features including BPRS, SAPS, SANS, and GAF scores. A *p*-value of 0.05 was considered to be statistically significant.

Multivariable regression models are used to establish the relationship between the major dependent variables (variation in IL17, BAFF, INF-γ, and TGF-β1) and demographic (gender and age), behavior (tobacco use, BMI has not been calculated), clinical (schizophrenia type and evolution, date of onset of the disease, previous treatment), standardized assessment scores, and biological (ESR and CRP) independent variables. The Kolmogorov–Smirnov Z test is used to reveal state markers, normalizing with treatment.

## 5. Conclusions

In summary, our study provides new insights into the immunomodulatory effects of antipsychotic drugs in schizophrenia. We demonstrated that IL-17 and BAFF are significantly altered after treatment, supporting their potential role as state markers or therapeutic targets. Our findings suggest that antipsychotic drugs may exert both pro- and anti-inflammatory effects on the cytokine network, depending on the cytokine involved. These findings open new perspectives for the development of personalized treatment approaches based on cytokine modulation. Further research is needed to explore the clinical relevance of these cytokine changes, their underlying mechanisms, and their potential role in the pathophysiology of schizophrenia.

## Figures and Tables

**Table 1:** Demographic and clinical data of schizophrenic patients.

Data	Patients (*n* = 63)
**Demographic characteristics**	
Age (years, mean ± SD)	36.81 ± 11.18
Sex: male (*n*, %)	35 (55.6)
**Clinical characteristics**	
Subtype (*n*, %)	
Undifferentiated subtype	33 (52.4)
Paranoid subtype	10 (31.7)
Disorganized subtype	20 (52.4)
**Age of onset of the disease** (years, mean ± SD)	24.41 ± 6.28
**Duration of illness** (years, mean ± SD)	12.37 ± 9.97
**Medication status on admission**	
Medication-naïve (*n*, %)	13 (20.7)
Medication-free (*n*, %)	50 (79.3)
**Antipsychotic treatment**	
First generation Antipsychotics	
Per os	18 (28.5)
Long-acting intramuscular injections	28 (44.5)
Second-generation antipsychotics	17 (27.0)

**Table 2:** Psychopathology measures of schizophrenia patients in the acute phase and after antipsychotic treatment.

Data of Standardized Assessment	Acute Phase (*n* = 63)	After Six Months (*n* = 52)	*z*	*p*
**BPRS**	−	34.53 ± 8.73	−6.251	<10^−3^
**PANSS**				
Global score	78.49 ± 13.16	52.32 ± 14.26	−6.241	<10^−3^
Positive subscale	21.70 ± 5.47	12.36 ± 5.15	−6.125	<10^−3^
Negative subscale	19.35 ± 6.11	13.92 ± 5.34	−5.636	<10^−3^
**General psychopathology**	37.40 ± 4.90	26.08 ± 5.90	−6.200	<10^−3^
**SAPS**	36.79 ± 13.01	18.47 ± 11.27	−6.182	<10^−3^
**SANS**	37.33 ± 15.23	24.66 ± 11.69	−5.758	<10^−3^
**GAF**	37.14 ± 5.65	51.60 ± 11.42	−5.666	<10^−3^

**Table 3:** Changes in cytokine levels following antipsychotic treatment.

Cytokine Concentrations	Acute Phase	After Six Months	*z*	*p*
	Median (IQR)	Median (IQR)		
IL-4 (pg/mL)	not detected	not detected	--	--
INF-γ (pg/mL)	19.00 (0.00–28.50)	0.00 (0.00–0.00)	−5.573	<10^−3^
TGF-β1 (ng/mL)	5120.00 (640.00–8960.00)	32.67 (00.00–60.50)	−5.637	<10^−3^
IL-17 (pg/mL)	84.00 (12.00–200.00)	0.00 (0.00–7.20)	−5.687	<10^−3^
BAFF(pg/mL)	740.00 (560.00–840.00)	857.50 (667.50–1073.75)	−2.395	0.017

**Table 4:** Cytokine markers association with drug treatment effects.

	Mean	Standard Deviation	Median	Z de Kolmogorov- Smirnov	*p*
Δ IL-17	−163.3729	299.65021	−82.0000	2.145	0.000 *
Δ TGF-β1	−6204.5415	6099.02682	−5702.5000	1.096	0.181
Δ IFN-γ	−17.2922	17.37129	−18.3500	1.344	0.054
Δ BAFF	277.9808	1221.53081	190.0000	2.177	0.000 *

Δ: Differences between six months and baseline values. * *p* < 0.005.

**Table 5:** Correlations between changes in cytokine serum levels and standardized assessment scores.

	Δ IFN-γ	Δ TGF-β1	Δ IL-17	Δ BAFF
Δ BPRS	r = 0.13 *p* = 0.33	r = 0.06 *p* = 0.62	r = 0.11 *p* = 0.42	r = −0.26 *p* = 0.058
Δ SAPS	r = 0.06 *p* = 0.67	r = 0.06 *p* = 0.64	r = 0.00 *p* = 0.95	r = −0.34 *p* = 0.011 *
Δ SANS	r = 0.39 *p* = 0.003 *	r = 0.08 *p* = 0.55	r = 0.07 *p* = 0.61	r = −0.89 *p* = 0.52
Δ GAF	r = −0.45 *p* = 10^−3^ *	r = −0.27 *p* = 0.04 *	r = −0.08 *p* = 0.56	r = 0.00 *p* = 0.99

Δ: Differences between six months and baseline values. r = Spearman’s correlation coefficients rho. * *p* < 0.005.

**Table 6:** Multivariable regression analysis model between changes in cytokine serum levels and scores.

Multivariable Regression Model		Standardized Beta Coefficients	*t*-Test	*p*
Δ IL-17	Δ BPRS	0.375	1.694	0.097
	Δ SAPS	−0.188	−0.878	0.384
	Δ SANS	−0.044	−0.236	0.815
	Δ GAF	0.086	0.472	0.639
Δ TGF-β1	Δ BPRS	0.040	0.186	0.854
	Δ SAPS	−0.123	−0.582	0.564
	Δ SANS	−0.173	−0.930	0.357
	Δ GAF	−0.395	−2.199	0.033 *
Δ INF-γ	Δ BPRS	−0.019	−0.100	0.921
	Δ SAPS	−0.194	−1.035	0.306
	Δ SANS	0.279	1.694	0.097
	Δ GAF	−0.405	−2.534	0.015 *
Δ BAFF	Δ BPRS	0.018	0.081	0.935
	Δ SAPS	−0.099	−0.455	0.651
	Δ SANS	−0.120	−0.622	0.537
	Δ GAF	−0.003	−0.015	0.988

Δ: Differences between six months and baseline values. * *p* < 0.005.

**Table 7:** Multivariable regression analysis model between changes in cytokine serum levels and demographic, behavior, clinical, and biological independent variables.

Multivariable Regression Analysis Model		Standardized Beta Coefficients	*t*-Test	*p*
Δ IL-17	Gender	−0.159	−0.522	0.604
	Age	0.036	0.204	0.840
	Age of onset of the disease	−0.037	−0.219	0.828
	Mode of disease onset	0.067	0.379	0.707
	Type of schizophrenia	0.195	1.077	0.288
	Trend mode	0.258	1.582	0.121
	CRP	−0.054	−0.332	0.742
	ESR	0.063	0.360	0.721
	Tobacco	−0.076	−0.261	0.796
Δ TGF-β1	Gender	0.077	0.268	0.790
	Age	−0.437	−2.594	0.013 *
	Age of onset of the disease	0.273	1.695	0.098
	Mode of disease onset	0.120	0.715	0.479
	Type of schizophrenia	0.105	0.614	0.543
	Trend mode	0.082	0.533	0.597
	CRP	0.089	0.575	0.568
	ESR	−0.208	−1.268	0.212
	Tobacco	−0.107	−0.385	0.702
Δ IFN-γ	Gender	−0.349	−1.167	0.250
	Age	0.078	0.448	0.656
	Age of onset of the disease	0.122	0.728	0.471
	Mode of disease onset	0.311	1.788	0.081
	Type of schizophrenia	−0.230	−1.293	0.203
	Trend mode	−0.024	−0.149	0.882
	CRP	−0.137	−0.851	0.400
	ESR	−0.007	−0.044	0.965
	Tobacco	0.181	0.629	0.533
Δ BAFF	Gender	−0.085	−0.278	0.782
	Age	−0.218	−1.223	0.228
	Age of onset of the disease	0.186	1.089	0.283
	Mode of disease onset	0.123	0.697	0.490
	Type of schizophrenia	0.136	0.748	0.459
	Trend mode	0.083	0.509	0.613
	CRP	−0.215	−1.305	0.199
	ESR	0.116	0.667	0.508
	Tobacco	−0.017	−0.056	0.955

Δ: Differences between six months and baseline values. * *p* < 0.005.

## Data Availability

The data presented in this study are available on request from the corresponding author.
